# Supplementary material for: Fluorinated oil-surfactant mixtures with the density of water: Artificial cells for synthetic biology
Source: PLoS One. 2022 Jan 20;17(1):e0252361. doi: 10.1371/journal.pone.0252361 (PMC8775225; doi:10.1371/journal.pone.0252361)
Supplement: S3 File — Additional figures and raw images. (DOCX) [file pone.0252361.s003.docx]

**Supporting information**

Fluorinated oil-surfactant mixtures with the density of water: artificial cells for synthetic biology

Roberto Laos^1,2*^, Steven Benner^1*^

^1^Foundation for Applied Molecular Evolution, Alachua, Florida, United States of America

Current address: 13709 Progress Blvd. Box 7, Alachua, Florida 32615, United States of

America

^2^Panamerican Biolabs LLC

Current address: 7226 NW 52nd Ter. Gainesville Florida 32653, United States of

America

* Corresponding authors: [manuscripts@ffame.org](mailto:manuscripts@ffame.org)

rlaos@ffame.org

Additional details, alternative protocols and data are available here.


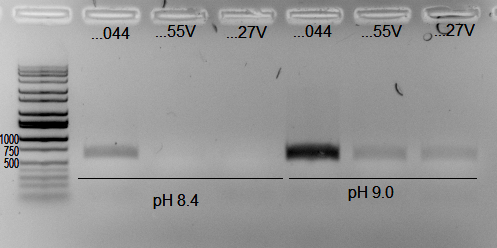


**S1 Figure. PCR in emulsion using different batches of Span^®^ 80.** Oil phase consists of: Span^®^ 80, 4.5%; Tween 80, 0.4%; Triton X-100, 0.05% in light mineral oil. Three different batches were prepared using Span80 from three different lots. Numbers on top of the lanes are the last three digits of the batch number: Batch 410044 - Fluka catalog 09569-50 mL; Batch MKBC7455V - Sigma catalog S6760-250mL; Batch MKBD4327V - Sigma catalog S6760-250mL.

Following [1], 1 x 10^8^ of induced *E. coli* cells were resuspended in 100 µL of buffer: 50 mM KCl; 10 mM TrisHCl/Tris base pH as indicated, 0.1% Triton X-100; 1.5 mM MgCl_2_, dNTPs 0.25 mM each; TMAC 50 µM; RNaseA 10 µg/mL; primers are 1 µM each. *E. coli* cells contain a gene that encodes for *Taq* DNA polymerase. The resuspended cells were added dropwise to 200 µL of oil phase with constant stirring at 1,000 rpm for 5 minutes. The emulsion was divided in PCR tubes and placed in a thermal cycler with the following program:

95 °C^3:30min^ [95 °C^30sec^; 64 °C^30sec^; 72 °C^30sec^]_20_ 72 °C^8:00min^

After PCR, the emulsions were spun down at 5,000 xg for 5 min and the top layer of oil was discarded. The remaining emulsion was broken with phenol/chloroform. The mixture was spun down again and the top aqueous layer was purified with Promega kit. Approximately 2/3 of the product was loaded in a 1% agarose gel and stained with ethidium bromide.

Oligos used as primers:

5’- CAGGAAGCAGCCATCACCTACTGGCCGCCTGAGCTCTAGC-3’

5'- CAGGAAGCAGCCATCACGCCCTGAACAGGCATGTTGAATGC-3’


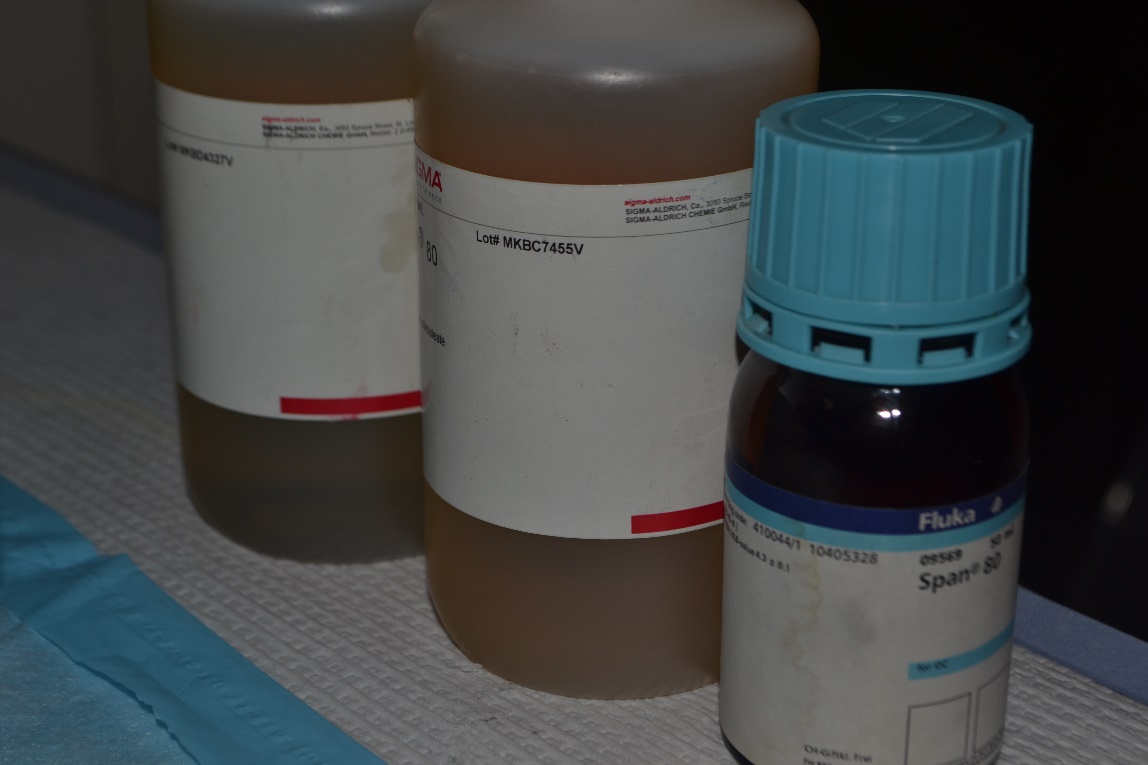


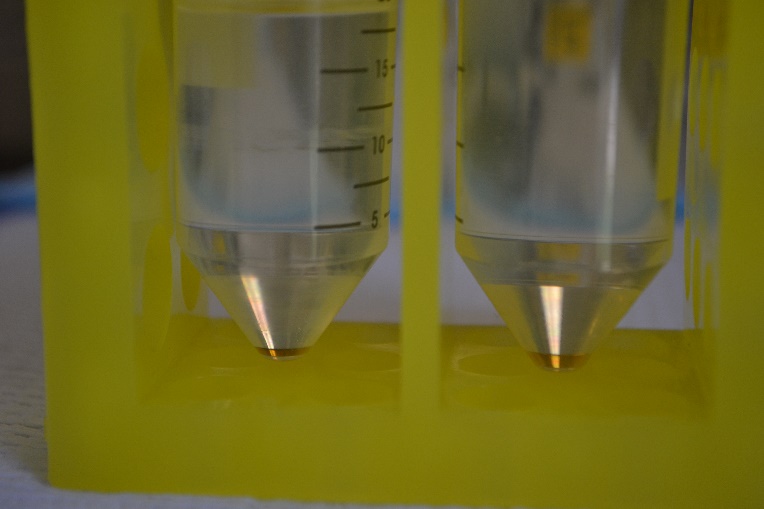


**S2 Figure. Dark precipitates observed in Span^®^ 80.** Top: dark precipitates observed in a bottle of Span^®^ 80 (left). Bottom: mineral oil solutions containing the surfactants: Span^®^ 80, 4.5%; Tween^®^ 80, 0.4%; Triton^™^ X-100, 0.05%. These oli/surfactant mixtures were first described in reference [1].





**S3 Figure. Example of synthesis of fluorinated nonionic surfactant a-R_2_.** Following reference [2] with some modifications: Hydrolysis of the chlorosilane (1) at room temperature for 2 hours to produce (2). Then (2) was reacted with (3) for 16 hours at 65 °C to produce (4), which was distilled (90-120 °C, 23 mmHg). Finally, hydrosilation of (4) and allyloxy(polyethylene oxide) (5) in the presence of potassium trichloro(ethylene) platinate (II) hydrate (6) and sodium acetate. The reaction proceeded for 4 hours at 80 °C. Finally, the amphiphilic molecule was filtered through neutral silica. A clear liquid was obtained.


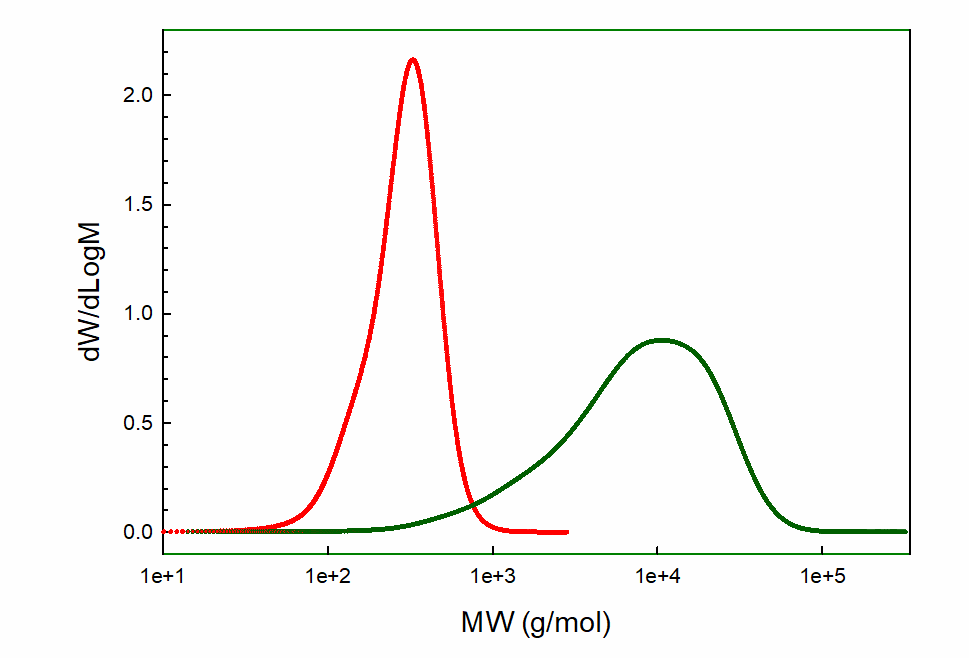


**S4 Figure. Overlay of molecular weight distribution of the two fractions separated by distillation after ring-opening polymerization following protocol 1.** The fractions show both an unimodal molecular weight distribution of Mp ~ 10320 g/mol for the high molecular weight fraction (green) and Mp ~ 320 g/mol for the low molecular weight fraction (red).


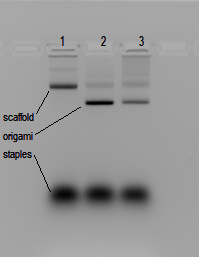


**S5 Figure. Origami fold in droplets using a QIAGEN TissueLyser II.** Electrophoresis in 2% agarose gel in 0.5 X Tris-borate EDTA buffer with ethidium bromide and 6 mM MgCl_2_. Line 1: mixture of scaffold and staples. Line 2: origami fold in aqueous phase only. Line 3: origami fold recovered from droplets. Emulsions were made by shaking using a QIAGEN TissueLyser II (15 Hz for 10 seconds followed by 17 Hz for 7 seconds) in a Snap-Cap Microcentrifuge Biopur™ Safe-Lock™ tube containing a 6 mm stainless steel bead.


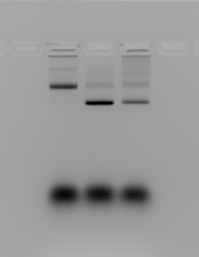


**S6 Figure. Raw image of S5 Figure.** Electrophoresis in 2% agarose gel in 0.5 X Tris-borate EDTA buffer with ethidium bromide and 6 mM MgCl_2_.Origami fold in droplets using a QIAGEN TissueLyser II.


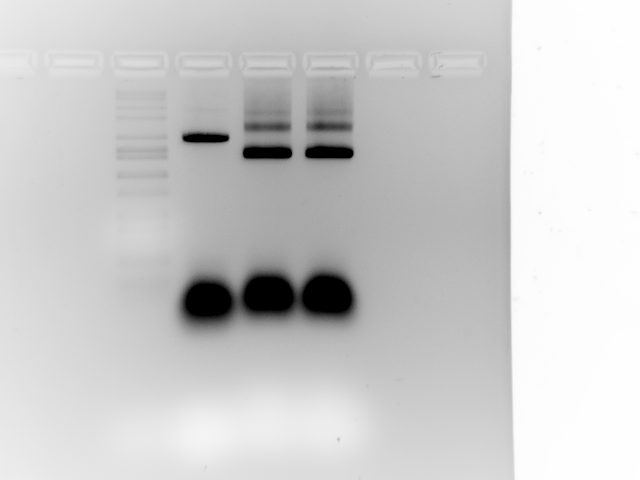


**S7 Figure. Raw image of Fig 11.** Electrophoresis in 2% agarose gel in 0.5 X Tris-borate EDTA buffer with ethidium bromide and 6 mM MgCl_2_ showing the formation of origami structures in droplets.


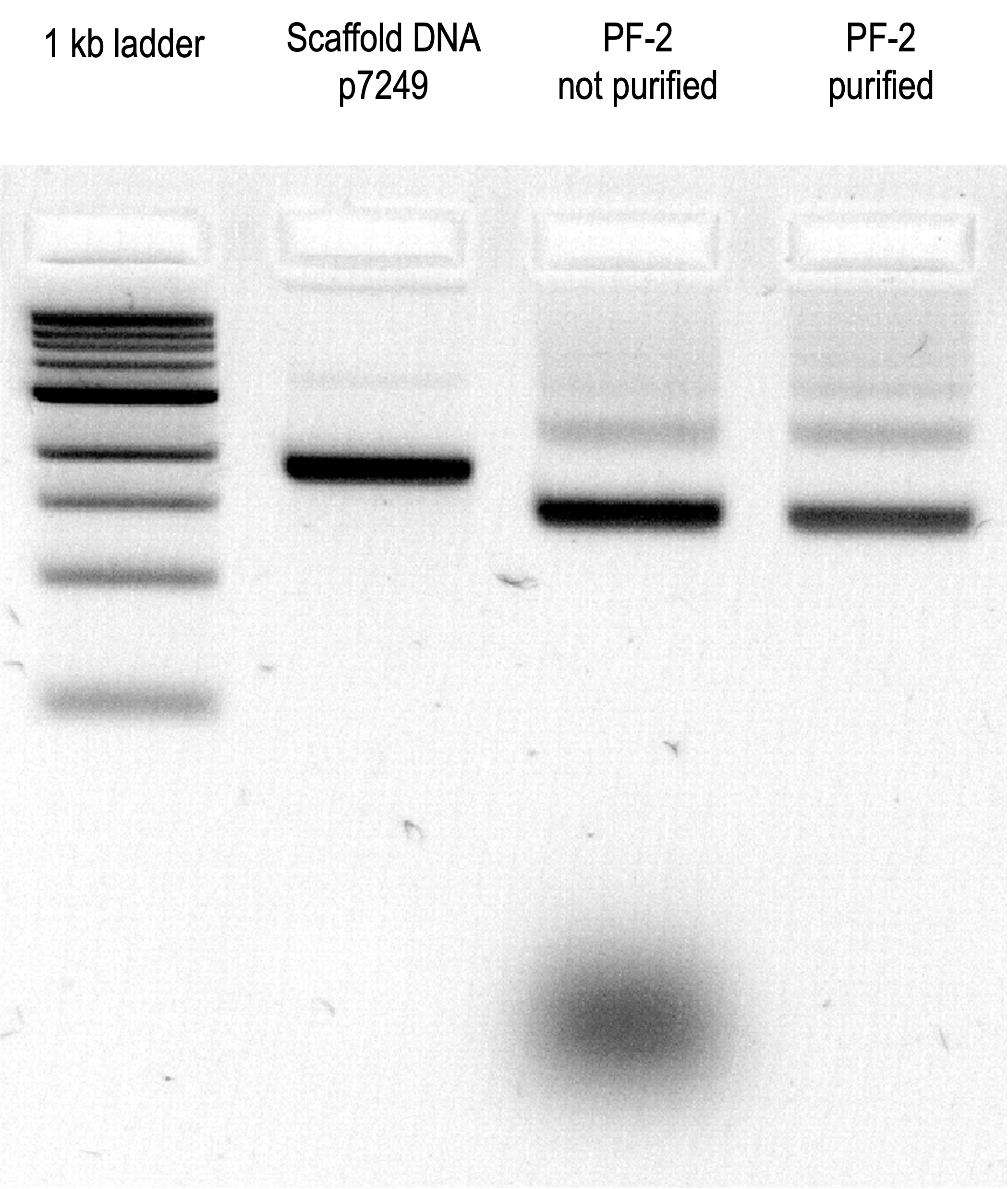


**S8 Figure. DNA electrophoresis of cuboid with large aperture from Tilibit Nanosystems.** DNA electrophoresis in 2% agarose, 0.5 X Tris-borate EDTA buffer with ethidium bromide and 6 mM magnesium acetate. The gel was post-stained with GelRed. The ladder is from NEB, the markers are: 10.0; 8.0; 6.0; 5.0; 4.0; 3.0; 2.0; 1.5; 1.0 and 0.5 kb base pairs. The scaffold runs near the 2.0 kb marker while the cuboid nanostructure runs near the 1.5 kb marker. The purified sample was analyzed by Transmission Electron Microscopy (S9 Figure). Figure provided by Tamara Aigner from Tilibit Nanosystems.


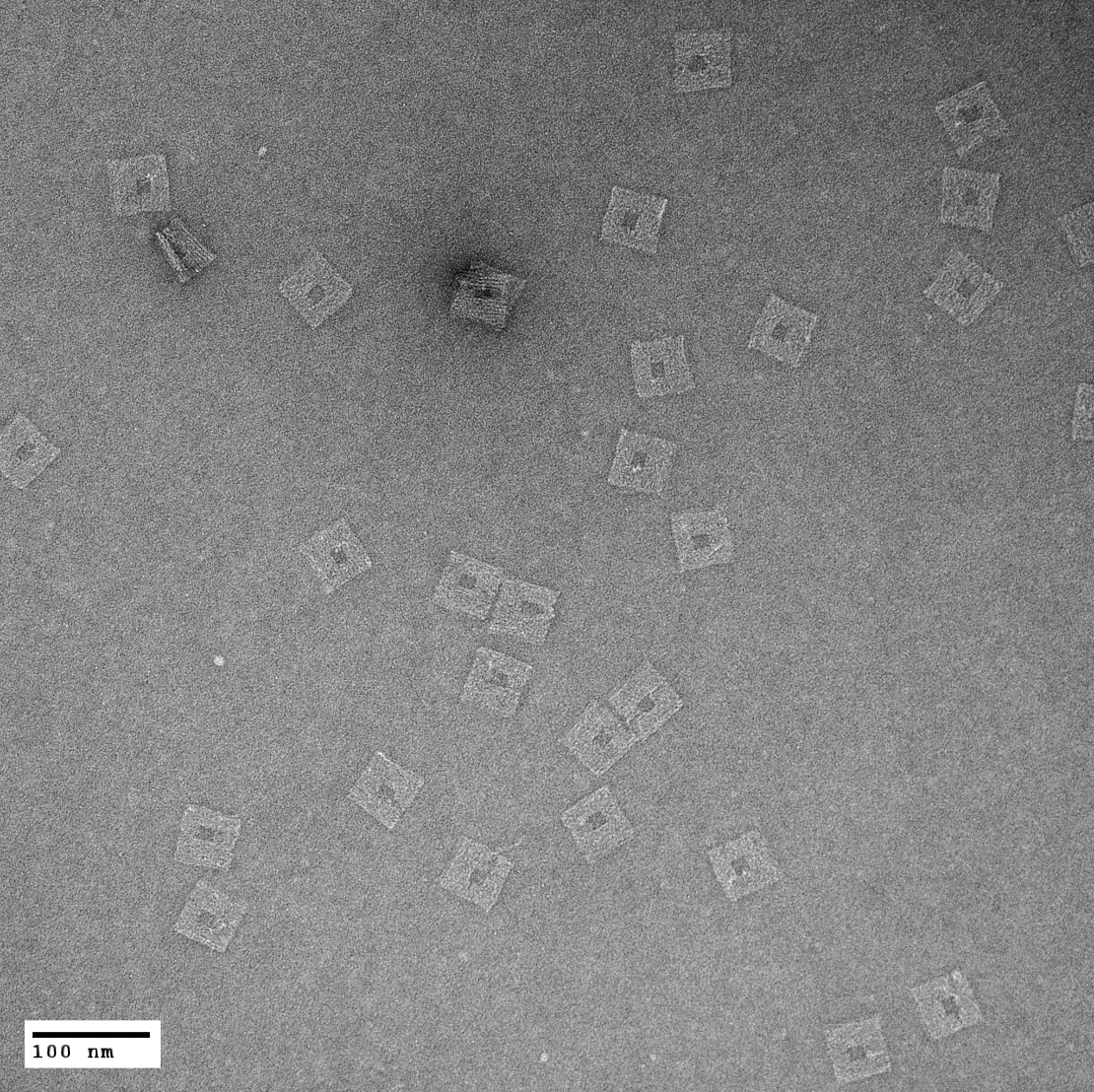


**S9 Figure. Transmission Electron Microscopy (TEM) of a correctly assembled DNA origami cuboid with large aperture from Tilibit Nanosystems.** The DNA origami structure forms a cuboid of dimensions 61 nm x 8 nm x 52 nm with an aperture of 9 nm x 15 nm. TEM image provided by Tamara Aigner from Tilibit Nanosystems.


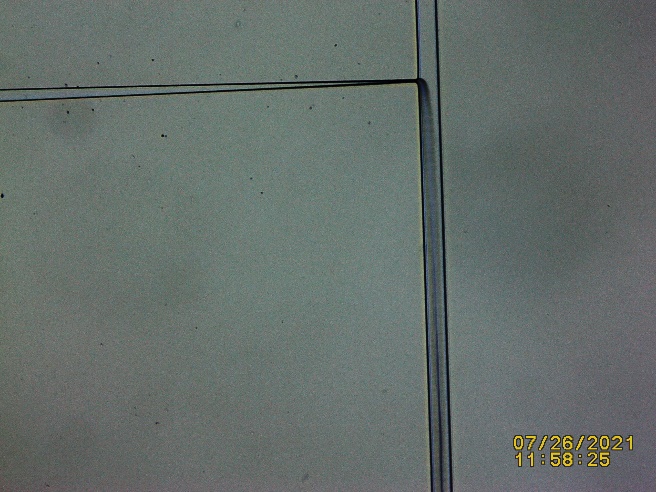

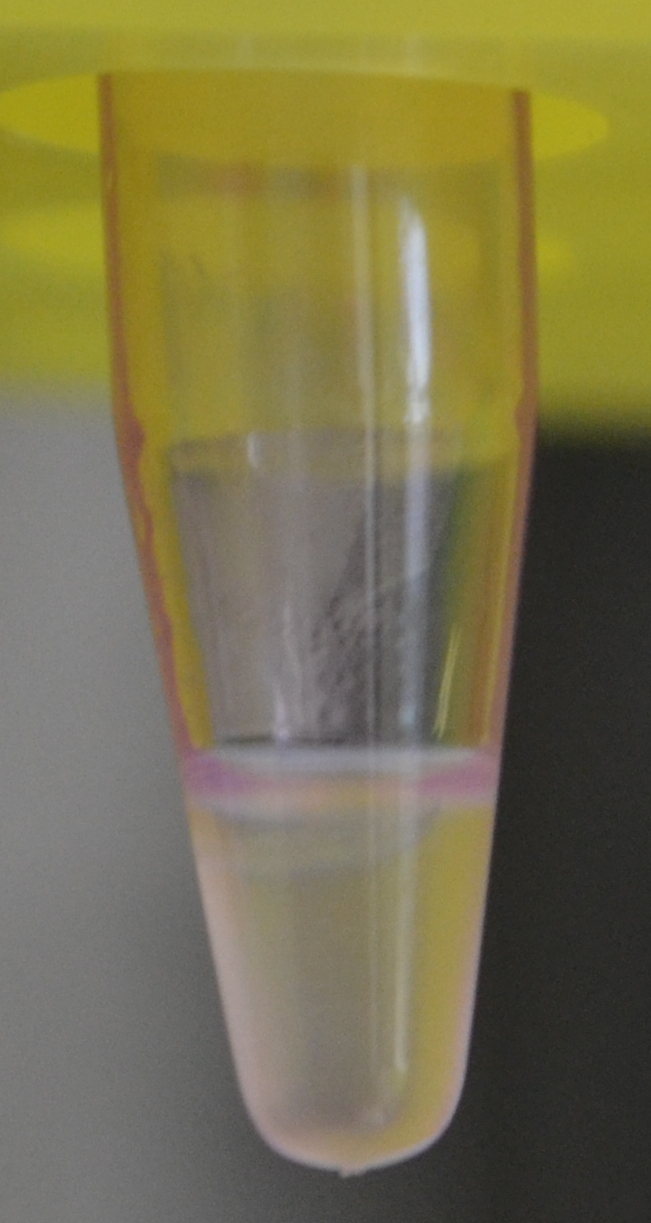


**S10 Figure. Droplets made using a microfluidic device (T-junction).** Left: microscope picture of the T-junction when the droplets are being generated. Right: picture of the droplets collected. Conditions: continuous phase was pumped at 3 µL/min; aqueous phase was pumped at 0.5 µL/min. Fluid B (distilled at 150 °C, 23 mm Hg) with 2% (v/v) surfactant (a-R_2_).

**S 1 Table.** **Sequences of oligos used as staples for origami structure PF-2 cuboid with large aperture.** Provided by Tamara Aigner.

| Staple sequences 5’ to 3’ |
| --- |
|  |
| ACCCTAAAGGGATTTTTT |
| TCTATCACCCTTATATGGTGGTTCCGAAAGTCACATCATGGAGGTCAGT |
| ACGTCAAGCCCGAGAGCAGGCGAAAATCGTGAGACCCATTGCTTGAAAG |
| TTTTTTAGAGTCCACTATTAAA |
| TTTTTTGCCCCCGATTTAACAGGGCGCTTTTTT |
| AAGCCGGCGCCGCGCTTAATGGACGAGCCGATTAACAAATGA |
| AGCGAAAACCATCAGAGTAAAAGAGTCTACATTTTTTGGCAGAGAACCC |
| GCGCTCGGCAAAATGGGCGATACGCAAATTAACCGAAAACGCCGACCAG |
| TGAGTGTGAACGTGAACATCACTTGCCTGTAATATCACCGCCTTTGCGT |
| TGTTCCAGTTTGGAACTTTTTT |
| GTAACCATTCCTCGTTAGAAACGTAAGA |
| CGCTGGTTGCCCTTCCAGAACTATCTAAAATATCTGTCAATA |
| TTTTTTAGTTGCAGCAAGCGGTCCA |
| TTTTTTGTACTATGGAGTCTTTAATTTTTT |
| ACGTGCTCCACACCCGAACGTGGCGAGATTTGGGGTTTATAA |
| ATTCACCATAGGGCGCTGGCAAGTGTAGATTTACAGACGCTCTCAAACC |
| TAATACACTGTTTGAAATCAAAAGAATAAGGGCGAAATACTT |
| AATCGTCGGCCACCCCCAAATCAAGTTTAAGGAAGGCTGCGC |
| TTTTTTACTATCGGCCACTAACAATTTTTT |
| TTTTTTCAGGAACGGTAC |
| GCCAGAATCCTGAGAGGAGGCACGTATACAATATT |
| TCAGTGATGATCAGAGCGGGACACCTTGCTGAACCAGAGGTG |
| CTTTGATCCGCCAGGGGCAACTGGTTTTTCTTTTCTCGGGAA |
| AGAACTCAATTTTTT |
| GGCTATTTTGCTTTCGCCGCTGAGCTTGACGGGGAAGCACTAAATCGGA |
| TTTGAATCCTAAAACCTGCAAACCTTTTACATCGGTTTTCAGGGAAGGG |
| ATACGTGATACCGAAGGCGGTAATAACGGATTCGCGAAATAA |
| TTCTGACGATGCCCGAACGTTCCAGAAGGAGCGGACACTGTA |
| TGGCCAATTAAAAGAATTCGAATCGCGCAGAGGCGTAAATCATTCCCTT |
| ATTGGGCCTGCATTGATAATAAGAAGATGATGAAAAACAATT |
| TTTTTTGGGGAGAGGCGGTGGCCCTGAGAGTTTTTT |
| TTTTTTTGCGCGAACTCCTGATTGTTTTTT |
| ACGAACCTCATATTCCTGATTCTACCATAGAAATTAGCCTTA |
| GTGCCAGGCCAGGGAGCTGATTTGCCCCATAGGGT |
| AATGAATCTCACATTAATTGCTAAAGTGTCATTTGGAGCCTA |
| CAGTATTAAAGCATGCTAAACAAGTGTTTCGAGGTGCCGTAA |
| TAGACTTTCAACAGAACAGGATTGTAGCAAAACCG |
| GAAGTATCATTTCAATTACCTTTTTTTAACGAGCCATCCGCT |
| TTTTTTCTAATAGATCAAGAAAACTTTTTT |
| TTTTTTGAGAGCCAGCAGAGGGATTTTAGATTTTTT |
| AAAATCTAACACCGCATCGCCGATGGCAATTCATCGAATAAT |
| CTCAATCTATTAAATCCTTAGCTTTGAATACCAAGATAACCT |
| AATATCTAATACCTGTCCATCGGCCCACTACGTGAGGAGCGG |
| TGGCAAATACAAACTTTGAGTAACATAGACAAAGGGACATTC |
| GAATTGAGGATTTAACCTGTCTCACTGCCCGCTTTACAACAT |
| ATTATCAACCAGCACTGAAAGATGGATTCGGTCACGGAAGAA |
| TTTTTTTGAGTGAGCTAACGGCCAACGCGCTTTTTT |
| TTTTTTTTTGGATTATTAACCTCCTTTTTT |
| ATCAAAAATAGTGAATTTATCGTAGGAATATAGAATAAACCAAGT |
| AATCGTCTTAAGACGCTGAGAAAAAACAGGGAAGC |
| AGAATACCCTATCATTTTGCGGAACAAAATTAATT |
| TAAAGCCTGTTTCCTGTGTGACCGGGTAAATAAGAAGAGATA |
| GTTTAACCCGACTTGCGGGAGCCGGTATATTTTAT |
| TGCTTCGGCTATTTTGCACCCAGAATAACATAATA |
| ATATATGATTTTATCCTGAATAAAATAGAATTAAC |
| ATGGAAAACGCTAACGAGCGTTTTTGTTACTCTAGGCCAAGC |
| AATTACCGAGCAAACATTTGAGGAAGGTAATATTATAGTAATGACTCCA |
| TTTTTTAAAATTAATAAATAAACATTTTTT |
| TTTTTTTACAGTAACAGTCAGTGCCACGCTTTTTTT |
| TAGGTCTGAGAGACTATAACTTCTAAGATATCATTCCAAGAA |
| CACAATTCAGGTCGTAACGTCGTAATTGAGCGCTATATCTTAGCGTGCT |
| TTTTTTCATGGTCATAGCTGGGGTGCCTAATTTTTT |
| TTTTTTGGCTTAGGTAAATCCAATTTTTTT |
| TTTCATCAAAATCATTAGAACATCAGATATTAAAAGCACAGA |
| GCATTAGGCTTAGAGCTATTAGAAACCAATTAATTCAGAGAT |
| GCAAGCAATTAGTTTAAAACACTGATTGATAAAACTCAAATA |
| CAGCCTTTACAGAGAGCTACATGAGTGATTACAAACAACTCG |
| TTTTTTGCCATATTAAGTTAAGCCTTTTTT |
| TTTTTTTTAGCGAACCTCGTCAGATGAATATTTTTT |
| AATCAAGAATCAGATCATTACCGCGCCCAGAGTCATTATTTG |
| ATTTGCCCAATCCACCGAGCTCGTTGTAAAACGACGATTAAG |
| AGTTACATACATTTCAAACATTAGAGCCTTAGGAGCTTGCTGGAGTAGA |
| CTGATGCTGGGTTATACCTTTTACTTCTAATATAATGATAGC |
| GTAAATGCGAGAAAAGAAACCAGACGACGACAATAAATATAAGGACATG |
| CATCGAGAACCAAGAAAAATAA |
| CCACCCTTGAACTGCCCCTTGAAAACATAGCGATAACGGGAG |
| TTGCATGGCAGATACCGAAGCACGCAATAATAACGTAAAGGTGGTTCAG |
| TTTTTTTCCCAGTCACGACGAATTCGTAATTTTTTT |
| TTTTTTCGCAAGACATAGTTAATTTTTTTT |
| GCAAGGCGGCCAGTAGGATCCAATTGTTGGAAGCAGTTGCGC |
| AGCATGTACATAAGTCCTGAAAAGCAAGCCGTTAT |
| ATTTACGCGGGTATGGCTTATGTTTTGAGCGTAGAGAGAAAC |
| TATCCCATCCTATGTTCAGCTA |
| CCTTTTTTCAGAGGAAAAATGCTTACCACAGTACAAATTATT |
| GAAATAGCAAAAGAACTGGCACAAACGTGGGAAGGGAAACCA |
| TTTTTTCAATAATAAGACTCCTTATTTTTT |
| TTTTTTGCTGTCTTTCCTACGCGAGGCGTTTTTTTT |
| ACCCACAAAACAATTTGGGTAATTACGCCAGCTGGAACTGTT |
| CGCGCCTGTTGGCAGAGGCATT |
| TTTTTTGGCCTCTTCGCTACGCCAGGGTTTTTTTTT |
| TTTTTTTCATCTTCTTGTGATAAATTTTTT |
| TAATTTATATCAACAATAGTTTTTCAAAATTTAATAGTACCGAGTATAA |
| GCTGCGCCGAAAGGGGGATCGAACAAAGACACCACGGCAACAATTCATA |
| TTCGAGCCAGTAAGTAGGGCTT |
| ACATACAGAATACCCAATAGCATATCAGAACGATTCTTTCCA |
| AGAAAATCAAAGACAAAAGGGATATTGAAGCCAGCATCGTAA |
| TTTTTTTTACGCAGTCAACCGATTTTTTTT |
| TTTTTTGTAATTCTGTCCAATCAATAATCGTTTTTT |
| TGAAATAAATAAACTAGTATCACCCTCAGAGCCGC |
| ATCGCCATATTAACCATTAGCAAGTCATCGGCATTTTCCGGTTAA |
| TAAGTTTAGTATCGGAATTATATATTCACAAACAA |
| GGCAAAGGCACTCCCGGAAATCTCATTAAAGCCAGCAGTAAG |
| TTTTTTCTTCTGGTGCCGGCGATCGGTGCGTTTTTT |
| TTTTTTTAAGGCGTTATCTTTTCATTTTTT |
| ATAGCAGTGAGCCACAGGTCAGACGATTTGGTAATAGTATTA |
| ATTAGAGCAATGAAGCGTCAGACTGTAGTTCGGAACTTGAGTTAACACT |
| ACCGACTCACCGTAATCAGACATTTTGTCGCAAAGTTACCAGAAAGTAA |
| TTTTTTGAGGGAGGGAGCGCAGTCTTTTTT |
| TTTTTTCAAATTCTTACCACAAAAGGTAAATTTTTT |
| AGCCAACGCCTGTTACCGGAAGCCCCCTTATTAGCAACCAGA |
| TGGTTTATAAAGGTGCCTCAGGTTTGAGGGGACGAGTGGGAA |
| CGACATTATGTTAGTGATTAAGAGCAAGAGAATTGTTTATCC |
| GTTTGCCAAATAAGCCGACCGGACCTAATATATTTAAGAACG |
| CCGTGCAACCGTAACGTCATATAGTTAGCGTAACGTAAATGA |
| TTTTTTGTAGATGGGCGCTTTCCGGCACCGTTTTTT |
| TTTTTTTAATCAAAAAGCCCGGAATTTTTT |
| CCGGAACGGTCATATCATAATTTAACAACGCCATT |
| TGCCCCCTACCAGGCGGATAAACATTTTTTCACGTCGATAGT |
| TGCCTATCGCGTTTGCCGGAAACGTCACCCAGCAAAGCATTG |
| AGAGGCCCGTAGCGACAGAATCAAGTTTACATGAAAAGTTTTGTACAAA |
| CGGATTGTCTGCCAGAAGATCCGCCATTCGCCAAA |
| TGGGATAACATTAAATGTGAGTAATTCGATTTTCTCGAAAGA |
| CACCCTCTCATTTTCAGGGATCGCCACCGGTTTAGCAACAAC |
| GAGCCGCGCCACCACGAGAGGGTTGATACTCAGGACTCAGAAGTAGCAA |
| TCCCTCACACCAGAAGAAAAAGCTCAACATAAGAGAACAACA |
| AACAGTGCGCCGCCAATCACCAGTAGCACCATTCTACCACCA |
| AGTGTACGGCCTTGCACCGTCATAGAAATATAAAACGAGGAA |
| ATACAGGGCATTCCACAGACAATTTTGCAACGCCAATCAGCT |
| TTTGATGCAAACGGCAACCCGTCGGATTCAATAGGTAAACAATACGAAG |
| CATGGCTATAAATCTATTCATCCAGCGC |
| TTTTTTTCTGAATTTGTTTTGTCGTTTTTT |
| TTTTTTCTCAGAGCCACCATATGCGTTATATTTTTT |
| CCAGAGCCCCGTATAAACACCCCAATAGGAACCCA |
| ACAGGAGTCAGTGCCCTATTAGATTAGCGGGGTTTCTCCAAA |
| AAGAGAAAGGAGCCAGCGGAGGGGTAAAATACGTAATCATCGCTTTGAA |
| TTTTTTCAGCTTTCATCAGGTCACGTTGGTTTTTTT |
| TTTTTTTAGGTGTATAACCGATATTTTTTT |
| CGTCTGGTTCGCATTAAATTTCAAATATTACCAAGGGAATAC |
| TTGCGAATAATACCTTGAGGACTAAAGAATGTTAC |
| AACAACTATGAGGAAGTTTCCTCGAAATTAAGGGAGCATAGG |
| TTTTTTTCTTTCCAGCTAAAACACTTTTTT |
| TTTTTTGAGCCACCACCCAGAACCGCCACCTTTTTT |
| GAGTTTCAGAAAGGAAAAAGGATTTCTTAAACAGCTCAATCA |
| CTACAACCAGTTTCTTTAATTGTATCACCTCTGAGACTCCTC |
| CATCGCCTTGCAGGCTCAGCAGGCTCATTATACCA |
| TGCGCCGTTCGGAACGAGGCGTCAAGAG |
| GAGGTGACTCCAAAGGATTAGTTCTGAAGCCTTTAACCATCG |
| CATTTTTAAAACAGCAACGGAAGATTCATCAGTTG |
| TTTTTTATTTTGTTAAAACCTTCCTGTAGCTTTTTT |
| TTTTTTATTCGGTCGTCAACGTAATTTTTT |
| CTGAGGCCACGCATCACCGTATAAGTATTCACCGG |
| AGAGGCATAGGTTTATCAGCTTGCTTTCTGACCAACCTGATAACAACAT |
| TTAGCTTCGTTGGGAAGAAAATGAGATGACCAGAAATTCATT |
| CCGCGACTTAATAAAACGAACAATAGCG |
| CGCGAAACCTAAAAGTATGGGGCCCTCA |
| ACCCCCAGAATACAACGTTAGATCTAAAACCGTTCAATGGAAAAGGTAA |
| TTTTTTTCATCTTTG |
| TTTTTTATCGGAACGAGGCCGCCACCCTCATTTTTT |
| CGGCTACCGTCACCGAGTTAAACCGGATATTCATT |
| ATTAAACTGAGAATGTCACCAAACGGGGGTTGAGGTTTGGGA |
| GCACCAACAAAGTAGAAGATTATAATCAGAAAAGCGCCTGAGATAACCC |
| GGCAAAAGCGATTATTAAATTCAATCATATGTACC |
| GACAAGAAGGCCGCACAATGATACCGCCGTGCCGT |
| TAATCTTGAGAAACGTTTAATACAGGTCAGGATTAATTAAGA |
| ACCTTCACAGACGGTTGATACTGAAAATTGCTCAG |
| CTGGCTGTACTTTTGCAAAAGTACTGCG |
| TGTACAGGCCAGAGACGATAAAAGAGGTCATTTTTATATATTGGCCGGA |
| TTTTTTAAACTAGCATGTGTAAACGTTAATTTTTTT |
| TTTTTTCAAAGCTGCCGAGAATGATTTTTT |
| GTGAATATTGTGAACCAGACCGGAAGCA |
| AGCAAACCCGGTTGGTATAAGTTGTTAATCAAAAACGAGTAA |
| AAGAGAACATAGTATATAATGCTGT |
| TTAATCAAGGCTTGTGCTTTAAACAGTT |
| TTCAACTGTCAGGAGCGGGATAGAGGCTTCAGAACAGCAAGC |
| TTGGGCTATCTACGCTGCTCCCTTTTTCAAAGGAATGTACCG |
| AGAGGAACCTTTAATTGCTCCTGAGTAA |
| CACTATCAGTCTGGAAAGGCTATCAGGT |
| TTACGAGGTCGATGAAGCTATTTTTGAGATCAACCG |
| TTTTTTTAACGCCAAGTTTTAAATTTTTTT |
| TTTTTTATTTTAAGAACTGCGAAAGACAGCTTTTTT |
| TATTACACCAGACGGGGGTAATTTAGACTGGATAG |
| GGTAGAAGATTTGTATGCCACCTTTCAAGCCTGTA |
| CACATTCAACTAATGCAGATACATTTTTT |
| GAATCCCTAGTCAGGGAAGCCTAATACTTTTGCGGGGCAAAG |
| GAATCGTGCAGGTATGTGTATCTTTATTTCAACGCTCCAATA |
| TTTTTTGCCGGAGAGGGTACGGTAATCGTATTTTTT |
| TTTTTTCCATAAATCAAAAATCCAGAAAATCATTCAACCCAAA |
| AGGTCTTTATCGCGACCAAAAACATTATAGCAATA |
| AAGCAAAGCGGATTCATAAATCGAGTAG |
| AAGATTCAAAAGGGCGTCCAAAAGTTTTACCAGGCACCGAAC |
| TGAGAAATTAAATGAAAATTTTTAGAACCTAATAG |
| GACAGTCCAAAATGTAGTATTCCACAGATGAACGG |
| AAATCACGTTGATTCGAACGAGTAGATTGAAAAGG |
| CTTCAAATACCCTGACTATTACCTCAAACCCTGAC |
| GAAGTTTACCATTAGATACATTTTTCAT |
| TTTTTTATGCAACTAATGGTCAATTTTTTT |
| TTTTTTCTTCAAAGCGAATTACCTTATGCGTTTTTT |
| TTTTGATAAACCAATAACGGAAATTGTG |
| GCTTAGAGCTTAATTATAACACATCAATATGATATGATCTAC |
| AGCGTGTCTGTTCTAGCTGATAAATTAATTTTTTT |
| TTTTTTTAAATCGGTTGTTTTTAATTCGAGTTTTTT |
| AAGCCTCAGAGCATAAAGCTTTTTT |
| AATTAGCAAAATTAGACCCTGCGAAAGAAACTCCA |
| AATCATACAGGCAAGAGAAGCCAAAAAGGAGAGTA |
| TAGTAGCATTAACAAAGGATACAATGCC |
| TGGCATCAATTCTACCTTCTGCCCAATCGCGGATGTCGTTTA |
| TTGGGGCGCGAGCTTAGTTTGCATTCCATGCTGAAAGAGCAAAGATTTA |
| TTTTTTAACCTGTTTAGCTATATTCGCAAAAGTACGTCAACATAAGGAA |

**References**

1. Ghadessy FJ, Ong JL, Holliger P. Directed evolution of polymerase function by compartmentalized self-replication. Proc Natl Acad Sci U S A. 2001;98(8):4552-7. doi: 10.1073/pnas.071052198. PubMed PMID: WOS:000168059700053.

2. Kobayashi H, Owen MJ. Nonionic Fluorosilicone Surfactants. Journal of Colloid and Interface Science. 1993;156(2):415-9. doi: 10.1006/jcis.1993.1131. PubMed PMID: WOS:A1993KT88300023.
